# A retrospective analysis of patient-specific factors on voriconazole clearance

**DOI:** 10.1186/s40780-016-0044-9

**Published:** 2016-04-19

**Authors:** Satoshi Dote, Maki Sawai, Ayumu Nozaki, Kazumasa Naruhashi, Yuka Kobayashi, Hirokazu Nakanishi

**Affiliations:** Department of Pharmacy, Kyoto-Katsura Hospital, 17 Yamadahirao-cho, Nishikyo-ku, Kyoto 615-8256 Japan; Faculty of Pharmaceutical Sciences, Doshisha Women’s College of Liberal Arts, Kodo, Kyotanabe-shi, Kyoto 610-0395 Japan

**Keywords:** Voriconazole, Pharmacokinetics, Therapeutic drug monitoring, Drug interaction, Inflammation

## Abstract

**Background:**

Voriconazole concentrations display a large variability, which cannot completely be explained by known factors. We investigated the relationships of voriconazole concentration with patient-specific variables and concomitant medication to identify clinical factors affecting voriconazole clearance.

**Methods:**

A retrospective chart review of voriconazole trough concentration, laboratory data, and concomitant medication in patients was performed. The concentration/dose ratio (C/D-ratio) was assessed as a surrogate marker of total clearance by dividing voriconazole concentration by daily dose per kg of body weight.

**Results:**

A total of 77 samples from 63 patients were obtained. In multiple linear regression analysis, increased C-reactive protein (CRP) level (*p* < 0.05) and decreased albumin (Alb) level (*p* < 0.05) were associated with significantly increased C/D-ratio of voriconazole, and coadministration with a glucocorticoid was associated with significantly (*p* < 0.05) decreased C/D-ratio of voriconazole (adjusted *r*^*2*^ = 0.31). Regarding CRP and Alb, receiver operating characteristic curve analysis indicated that increased CRP level and decreased Alb level were significant predictors of toxic trough concentration of voriconazole. For CRP, area under the curve (AUC) and cutoff value were 0.71 (95 % confidence interval (CI), 0.57–0.86, *p* < 0.01) and 4.7 mg/dl, respectively. For Alb, AUC and cutoff value were 0.68 (95 % CI, 0.53–0.82, *p* < 0.05) and 2.7 g/dl, respectively. A significant difference was seen in voriconazole trough concentration between patients with hepatotoxicity and those without (5.69 μg/ml vs 3.0 μg/ml, *p* < 0.001).

**Conclusion:**

Coadministration of glucocorticoid and inflammation, reflected by elevated CRP level and hypoalbuminemia, are associated with voriconazole clearance. We propose that early measurement of voriconazole concentration before the plateau phase will lead to avoidance of a toxic voriconazole level in patients with elevated CRP level and hypoalbuminemia, although further studies are needed to confirm our findings.

## Background

The triazole antifungal voriconazole is widely used in the treatment of invasive fungal infections, such as invasive aspergillosis, candidemia, and pulmonary cryptococcosis, except for zygomycosis [1–4]. Voriconazole exhibits a nonlinear pharmacokinetics profile and is metabolized primarily by the cytochrome P450 family enzyme CYP2C19 and, to lesser extent, CYP3A4 and CYP2C9 [1–4]. Allelic polymorphisms of CYP2C19 have been shown to be the most important determinants of the clearance efficiency of vorizonazole [3, 5]. In Japanese, the rate of poor metabolizers of CYP2C19 has been shown to be 15–20 %, a higher figure than that of other races [6]. This raises the necessity of blood concentration measurement for Japanese to monitor efficacy and toxicity in the clinical setting. There have been some reports on the relationships between efficacy and toxicity of voriconazole and its plasma concentrations. Target trough concentration of therapeutic response is > 1–2 μg/ml [1, 3, 4, 7–9], and prophylaxis is > 1.5 μg/ml [3]. To avoid side effects, such as visual disturbance, neurotoxicity, and hepatotoxicity, the target trough concentration is < 4–6 μg/ml [1, 3, 4, 7–10]. Invasive fungal infections commonly appear among immunocompromised patients receiving concomitant medication for primary disease and/or bacterial infection. Many of them have critically ill conditions and polypharmacy, which possibly alter voriconazole concentration and clearance. Accordingly, to identify clinical factors affecting voriconazole clearance, we investigated the relationships of voriconazole concentration with patient-specific variables and concomitant medication.

## Methods

### Patient enrollment and data collection

Patients who received voriconazole orally and had at least one voriconazole trough concentration measurement during therapy at Kyoto-Katsura Hospital, Japan, between June 2010 and October 2015 were eligible for inclusion. Patient-specific variables were collected retrospectively including sex, age, body weight, body mass index (BMI), laboratory data, purpose of voriconazole administration, and diagnosis and treatment department. Laboratory data included serum albumin (Alb), creatinine clearance (Ccr), aspartate aminotransferase (AST), alanine aminotransferase (ALT), total bilirubin (T-Bil), alkaline phosphatase (ALP), γ-glutamyl transpeptitase (γGTP), and C-reactive protein (CRP). Ccr was estimated by using the Cockcroft-Gault Formula [11]. Laboratory data at voriconazole trough concentration measurement were used for analysis. Daily dose, voriconazole trough concentration, the number of days between blood sample collection and initial dose were collected. Patients were excluded if: (a) they received voriconazole intravenously even once to avoid unknown covariates between orally and intravenously; (b) they received concomitant rifabutin, rifampicin, carbamazepine, phenytoin, or antiretroviral drugs; (c) they required dialysis since the uremic substances, which should accumulate in the blood of those patients, may alter the pharmacokinetics of voriconazole; or (d) the number of days between blood sample collection and initial voriconazole administration with loading dose is 2 or less, or that without loading dose is 4 or less, because the concentration did not reach a steady state [3, 4]. Affect of concomitant glucocorticoid (over 5 mg equivalent prednisolone as a daily dose) and macrolide was analyzed in all patients, because those medications can interact with voriconazole pharmacokinetically [9, 12, 13]. Cases in which the voriconazole dose was changed, or the above-mentioned concomitant medications were started or discontinued, were regarded as separate cases. Visual disturbance such as photophobia, blurred vision, neurotoxicity such as drowsiness, insomnia, hallucinations, disturbance of consciousness, and hepatotoxicity were evaluated as adverse events. Hepatotoxicity was defined as ≥ grade 2 in results of liver function tests (AST, ALT, T-Bil, ALP, and γGTP) within 14 days after initiation of voriconazole administration or changing voriconazole dosage, according to *Common Terminology Criteria for Adverse Events (CTCAE), version 4.0*, since some of ALP and γGTP values in the patients were over normal range (about grade 1) before the voriconazole therapy. We used voriconazole concentration at the time of occurrence of toxicity in the patients with adverse event, whereas the maximum concentration in the patients without adverse event for the analysis of relationship between adverse events and voriconazole concentration. All voriconazole concentration data were collected from a central referral laboratory (SRL Inc., Tokyo, Japan). A validated high-performance liquid chromatography (HPLC) assay was used to measure voriconazole concentrations [14].

The study was performed in accordance with the Declaration of Helsinki and its amendments, and the protocol was approved by the Ethics Committee of Kyoto-Katsura Hospital.

### Analysis of voriconazole pharmacokinetics

The concentration/dose ratio (C/D-ratio) was used as a surrogate marker of total clearance by dividing voriconazole concentration by daily dose per kg of body weight.

### Statistical analysis

Univariate linear regression analysis was used to estimate relationships between C/D-ratio of voriconazole and patient-specific continuous variables, and the unpaired t test was used to compare voriconazole concentration and C/D-ratio of voriconazole between two independent groups. Multiple linear regression analysis was applied to identify factors that contribute to the variability in C/D-ratio of voriconazole; a step-down selection model was obtained using stepwise regression with a *p* value of 0.25 as the inclusion criterion. The diagnostic properties and cutoff values of independent variables by multiple linear regression analysis were cross-sectionally evaluated by a receiver operating characteristic (ROC) curve. Outcome for diagnostic properties was voriconazole trough concentration exceeding 4 μg/ml, defined as a toxic level according to the results of meta-analysis by Hamada et al. [8]. JMP®9 software was used for all analyses and a *p* value less than 0.05 was regarded as statistically significant.

## Results

### Patient characteristics

A total of 77 samples from 63 patients were obtained in the present study (Table 1). Eligible patients were elderly, with a mean age of 70.8. Mean daily maintenance dose and mean body weight were 273 mg and 49.7 kg, respectively. The mean dosage is close to, but slightly lower than the recommend daily oral dose on the package insert [15] for patients with a body weight ≥ 40 kg (300–400 mg). This may be caused by the suggestion by the clinical pharmacist to the prescription doctors to reduce the voriconazole dosage, since the pharmacist had experienced higher concentration of voriconazole in patients with higher CRP values, empirically. Twenty-eight patients received glucocorticoid as a concomitant medication and most of them (23. 82 %) received prednisolone.

**Table 1:** Patient characteristics

	Mean (SD)
Male/Female	50/13
Age (yr)	70.8 (11.0)
Body Weight (kg)	49.7 (9.5)
Body Mass Index (kg/m^2^)	19.0 (2.9)
Albumin (g/dl)^a^	2.7 (0.7)
Creatinine clearance (ml/min)^a^	67.4 (31.9)
AST (IU/L)^a^	32.9 (28.6)
ALT (IU/L)^a^	32.9 (35.9)
ALP (IU/L)^a^	386.6 (409.2)
γGTP (IU/L)^a^	103.7 (173.1)
T-Bil (mg/dl)^a^	0.6 (0.8)
C reactive protein (mg/dl)^a^	5.6 (6.4)
Daily dose (mg/day)^a^	273 (89.8)
Trough concentration (μg/ml)^a^	3.2 (2.3)
Trough concentration collection date (after initial dose)^a^	
with loading dose	9.7 (13)
without loading dose	11.1 (9.5)
Purpose of voriconazole administration prophylaxis/treatment	3/60
Diagnosis and treatment department	
Hematology/Pulmonology/Rheumatology/Other	41/17/4/1
Concomitant medication^a^	
Glucocorcicoid	28
Macrolide Antibiotics	2

^a^A cumulative total of 77 samples

These data were collected at the time of initial voriconazole administration

### Factors affecting voriconazole concentration and clearance

**Fig. 1:**
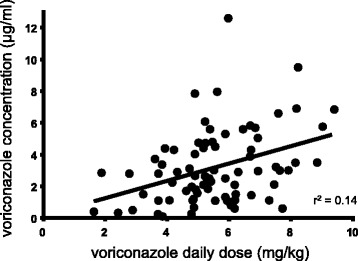
Relationship between voriconazole dosages per patient weight and voriconazole trough concentration. Each point represents a measurement. The linear regression curve is presented with coefficient of determination (r^2^)

**Fig. 2:**
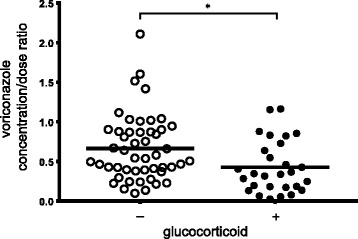
Comparison of mean voriconazole concentration/dose ratio. A scatter plot of voriconazole concentration/dose ratio from 77 samples for 63 patients without coadministration of glucocorticoid (*n* = 49) and with coadministration of glucocorticoid (*n* = 28). Solid line means mean

Significant correlation was found between administered dosage and trough concentration of voriconazole (*r*^*2*^ = 0.14, *p* < 0.001), (Fig. 1). Mean (SD) C/D-ratio significantly decreased from 0.66 (0.42) to 0.43 (0.33) by concomitant administration with glucocorticoid (*p* = 0.01) (Fig. 2). Effect of concomitant macrolide on C/D-ratio was not conducted because only two patients was administered erythromycin. Significant correlations were found between C/D-ratio of voriconazole and Alb (*r*^*2*^ = 0.16, *p* < 0.01), CRP (*r*^*2*^ = 0.26, *p* < 0.0001). Alb, CRP, and age (*p* = 0.07) were treated as continuous variables and glucocorticoid use was treated as a dichotomous variable in multiple linear regression analysis. Increased CRP level and decreased Alb level were significantly associated with increased C/D-ratio of voriconazole, and coadministration with a glucocorticoid was significantly associated with decreased C/D-ratio of voriconazole (adjusted *r*^*2*^ = 0.31) (Table 2). We did a post-hoc analysis of the effect of coadministration with a glucocorticoid on interpatient variability of voriconazole concentration in three patients (Table 3). C/D-ratio was considerably decreased by glucocorticoid administration in all three patients.

**Table 2:** Factors associated with a significant change in vorizconazole clearance identified from multiple linear regression analysis

		95 % Confidence interval			
Model term	Coefficient	Lower	Upper	Variance inflation factors	*p* value
Albumin (g/dl)	−0.19	−0.36	−0.03	1.55	<0.05
C reactive protein (mg/dl)	0.02	0.004	0.04	1.51	< 0.05
Glucocorticoid administration	−0.25	−0.45	−0.05	1.18	< 0.05

**Table 3:** Change of voriconazole concentration and clearance about the patient with and without glucocorticoid administration

Patient	Case. 1		Case. 2		Case. 3	
	Pre	Post	Pre	Post	Pre	Post
Glucocorticoid	mPSL 62.5 mg bid	-	-	DEX 20 mg	-	PSL 20 mg
Daily dose (mg/day)	300	300	200	150	400	200
Trough concentration (μg/ml)	4.03	7.85	3.37	0.5	3.22	0.1
C/D-ratio	0.82	1.6	0.88	0.17	0.43	0.03

Abbreviations: *mPSL* methylprednisolone, *DEX* dexamethasone, *PSL* prednisolone, *bid* twice daily

### Cutoff values of factors causing toxic level of voriconazole concentration

**Fig. 3:**
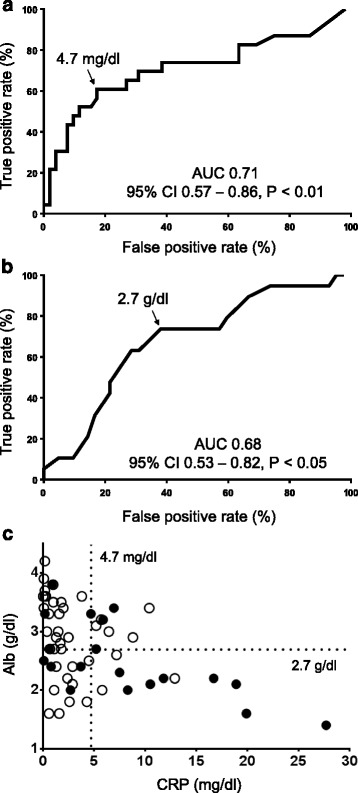
Receiver operating characteristic (ROC) curves for predicting risk of toxic voriconazole level on the basis of CRP (**a**) and Alb (**b**); relationships of toxic voriconazole level with CRP and Alb (**c**). The true-positive rate represents the proportion of true positives that are correctly classified as positive. The false-positive rate represents the proportion of true negatives that are incorrectly classified as positive. True-positive rate = true positives/(true positives + false negatives). False-positive rate = false positives/(false positives + true negatives). *Closed circles* represent patients with toxic voriconazole level and *open circles* represent patients without toxic voriconazole level. 95 % CI, 95 % confidence interval

Multiple linear regression analysis indicated that CRP and Alb were the factors affecting voriconazole clearance. ROC curve analysis indicated that increased CRP level and decreased Alb level were significant predictors of toxic trough concentration of voriconazole: A) CRP - area under the curve (AUC) 0.71 (95 % confidence interval [CI], 0.57–0.86, *p* < 0.01), cutoff value 4.7 mg/dl (sensitivity 60.9 %, specificity 82.7 %); B) Alb - AUC 0.68 (95 % CI, 0.53–0.82, *p* < 0.05), cutoff value 2.7 g/dl (sensitivity 61.9 %, specificity 73.7 %) (Fig. 3).

### Adverse effects

**Fig. 4:**
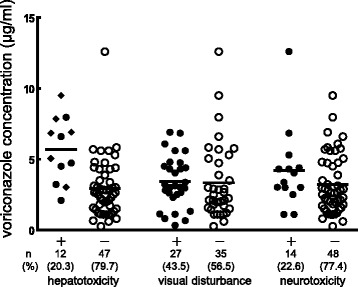
Relationships between voriconazole concentration and toxicities. A scatter plot of voriconazole concentrations from all 63 patients. *Closed circles* represent patients with toxicity and *open circles* represent patients without toxicity. *Closed diamond* represents patients with Grade 3 hepatotoxicity. *Solid line* means mean. One patient was not assessed for visual disturbance and neurotoxicity because of the administration of a sedative. Four patients were not assessed for hepatotoxicity because of liver dysfunction due to primary disease. A significant difference was seen in voriconazole mean trough concentration between patients with grade 2-3 hepatotoxicity and those without: 5.69 (SD 2.27) vs. 3.0 (SD 2.07), *p* < 0.001. No significant difference was seen in voriconazole mean trough concentration between patients with other adverse events and those without: visual disturbance - 3.48 (SD 1.79) vs. 3.44 (SD 2.7), *p* = 0.94; neurotoxicity - 4.2 (SD 2.85) vs. 3.27 (SD 2.11), *p* = 0.18

Incidence of hepatotoxicity, visual disturbance, and neurotoxicity were 20.3, 43.5, and 22.6 %, respectively. Twelve episodes of hepatotoxicity were observed: grade 2, six; grade 3, six. Summary of hepatotoxicity were: grade 2 elevation of ALT, one; of ALP, two; of γGTP, four; grade 3 elevation of ALT, one; of ALP, two; of γGTP, six. The voriconazole trough concentration were significantly higher in patients with grade 2–3 hepatotoxicity than in patients without, whereas no significant difference was seen in visual disturbance and neurotoxicity (Fig. 4).

## Discussion

The results of the present study have shown that CRP elevation and hypoalbuminemia decreased voriconazole clearance and that concomitant glucocorticoid administration increased voriconazole clearance. In multiple linear regression analysis, we did not detect the presence of multicolinearity between CRP and glucocorticoid administration (variance inflation factors < 2), suggesting that CRP and concomitant glucocorticoid administration should be independent of each other in affecting voriconazole clearance. Although these two factors are independent, we speculate these factors might be associated with alteration of metabolism of voriconazole; elimination of voriconazole is mainly by metabolism of CYP2C19 dominantly and of CYP3A4 and CYP2C9 being involved to a much lesser extent [1–4].

Michael et al. retrospectively investigated therapeutic drug monitoring of voriconazole in a cohort of 201 patients [9]. They identified by multiple linear regression analysis that the use of a systemic glucocorticoid was associated with significantly reduced voriconazole blood concentrations. They hypothesized that glucocorticoid up-regulated CYP2C19 and CYP3A4 expression since some in vitro [16, 17] and in vivo [18, 19] studies have shown that glucocorticoid can bind to and activate the pregnane X receptor, one of the nuclear receptors which plays a role in inducing cytochrome P450 expression, including CYP2C19 and CYP3A4, resulting in the increase of metabolism by the enzymes.

Another study has shown that voriconazole clearance significantly decreased in a limited number of patients with CYP2C19 poor metabolizer and heterozygous extensive metabolizer when coadministered with erythromycin, a known CYP3A4 inhibitor [12]. In our study, of the three patients who administered concomitant glucocorticoid, C/D-ratio of one patient was considerably increased by discontinuation of glucocorticoid (Table 3, Case 1), and C/D-ratios of the two patients were considerably decreased by start of glucocorticoid (Table 3, Cases 2 and 3). They might be poor metabolizer of CYP2C19, resulting in the prominent increase of voriconazole metabolism due to CYP3A4 induction, although we did not perform the allele analysis of any gene of the patients.

Zordoky et al. reported that nuclear factor-κB plays a crucial role in the regulation of cytochrome P450 through several mechanisms and this role can explain the altered cytochrome P450 regulation in many conditions [20]. They suggested that in inflammation status, nuclear factor-κB-repressing factor is activated and cytochrome P450 expression is decreased. A significant decrease in voriconazole clearance observed in patients with elevated CRP level in our study. Other studies reported the inflammation is associated with higher voriconazole trough concentrations [13, 21], congruent with our results. Therefore, higher voriconazole trough concentrations in inflammatory patients might be related to the above mechanism, since elevated CRP level and hypoalbuminemia are regarded as markers for inflammatory status.

In addition, we undertook an ROC analysis for prediction of risk of toxic voriconazole level on the basis of CRP and Alb. The results suggest that CRP and Alb should be useful for screening of toxic voriconazole level. In particular, in most cases, voriconazole concentrations reached toxic levels when the CRP and Alb values of the patients were over the cutoff values described below (Fig. 3c). Consequently, careful monitoring of voriconazole concentration and side effects are required for patients with both elevated CRP level and hypoalbuminemia. From our analysis, the cutoff values of CRP and Alb were 4.7 and 2.7 respectively. Statistically, these cutoff values are useful for prediction of toxic voriconazole level, although the value of 4.7 of CRP is less clinically important, since the value is common level of infectious diseases. Voriconazole concentrations in patients with hepatotoxicity were significantly higher than those without. In addition, the concentrations of voriconazole in 75 % of patients with hepatotoxicity were over 4 μg/ml. This result suggests that voriconazole trough concentration over 4 μg/ml should be avoided, supported by previous reports [8, 10].

A limitation of the present study is that CYP2C19 polymorphism was not available. However, Geist et al. found an enormous (88 %) interpatient variability in voriconazole trough concentrations, although none of the patients were classified as a CYP2C19 poor metabolizer. They noted that the high variability resulted from contributing factors, including changes in drug absorption, co-medication, and disease status [22]. Further studies are needed to clarify contributing factors in patients with known genetic polymorphisms of the cytochrome P450 isoenzymes.

## Conclusion

We evaluated patient-specific factors affecting voriconazole clearance and showed that increased CRP level and decreased Alb level were significant predictors of decreased voriconazole clearance, and coadministration of glucocorticoid was found to be a significant predictor of increased voriconazole clearance. It is not practical to examine CYP2C19 alleles due to the cost, so it is difficult to consider CYP2C19 polymorphism in therapeutic drug monitoring in routine practice. Therefore, we propose that early measurement of voriconazole concentration before the plateau phase should lead to the avoidance of toxic voriconazole level in patients with elevated CRP level and hypoalbuminemia.
